# Granulocyte Colony-Stimulating Factor for Treatment of Patients with Chronic Traumatic Brain Injury: A Preliminary Pre-Post Study

**DOI:** 10.3390/brainsci11111441

**Published:** 2021-10-29

**Authors:** Tsung-Lang Chiu, Ya-Jung Wang, Tze-Wei Chang, Shinn-Zong Lin, Sheng-Tzung Tsai

**Affiliations:** Department of Neurosurgery, Hualien Tzu Chi Hospital/Tzu Chi University, Hualien 970, Taiwan; poluschiou@gmail.com (T.-L.C.); guagua1019@gmail.com (Y.-J.W.); kenny702187@gmail.com (T.-W.C.); shinnzong@yahoo.com.tw (S.-Z.L.)

**Keywords:** G-CSF, traumatic brain injury, outcome

## Abstract

Chronic traumatic brain injury (TBI) can cause permanent disability and thereby negatively affect patients, families, and society. Currently, there is no effective treatment for patients with chronic TBI. One possible option is granulocyte colony-stimulating factor (G-CSF), which has potential neuroregenerative and neuroprotective effects through its ability to mobilize hematopoietic stem cells and increase neurogenic growth factor levels. Previous studies have shown that G-CSF administration is safe for patients with neurological diseases such as stroke and dementia. The present study aimed to explore the safety and efficacy of G-CSF use in patients with chronic TBI. Methods: 38 patients with chronic TBI were administered 3-day rounds of G-CSF (10 μg/kg per day) once a month for 6 months. These patients were clinically evaluated using the modified Rankin scale (mRS) and Karnofsky Performance Score (KPS). Laboratory measures of the leucocyte counts and differential count percentage were also assessed. Results: At the 6-month follow-up, further assessment showed that patients tolerated the treatment well with only mild and transient side effects being observed. Further clinical evaluation showed significant improvements in mRS and KPS after G-CSF treatment. Laboratory results also confirmed the action of the medication, with increased leukocytosis and band forms. Conclusions: The results suggest that 6-month chronic G-CSF treatment is safe for patients with chronic TBI and may provide clinical benefits and neurological improvements. The adverse effects of the treatment, however, are transient and usually tolerable. Thus, these preliminary findings suggest that future clinical trials of G-CSF use in patients with chronic TBI are warranted.

## 1. Introduction

Traumatic brain injury (TBI) is one of the leading causes of mortality and disability worldwide. At the subacute stage, rehabilitation usually plays an important role in improving the neurological disability and functional status of patients with TBI; however, for those with chronic TBI, i.e., having suffered the injury more than 6 months ago, the extent of the functional recovery can be limited even after intense rehabilitation. Indeed, patients with chronic TBI usually suffer from severe cognitive and motor deficits that reduce their ability to perform daily activities and thereby their quality of life. Several pharmacological treatments have been studied with the aim of enhancing recovery during chronic TBI; however, such trials have thus far failed to show significant benefits or amelioration of disability [1]. An alternative neurorestorative and/or reparative strategy could involve using stem cells or agents that release endogenous stem cells to treat chronic TBI.

Granulocyte colony-stimulating factor (G-CSF), a member of the cytokine family of growth factors, mobilizes hematopoietic stem cells (CD34+) from the bone marrow into the peripheral blood. G-CSF is used routinely to treat neutropenia and for bone marrow reconstitution. However, the mobilized hematopoietic stem cells have demonstrated that G-CSF might have neuroprotective and regenerative effects [2]. The mechanisms by which G-CSF improves neural plasticity also involve its anti-inflammatory and anti-apoptotic effects, both of which have been implicated in the pathophysiology of chronic TBI [3,4,5,6]. Additionally, the G-CSF receptor has been found throughout the central nervous system and G-CSF is able to cross the blood–brain barrier [7]. Importantly, G-CSF can mobilize stem cells and facilitate their integration over injured neurons [8,9,10]; thus, G-CSF has the potential for use in clinical applications, including the treatment of chronic stroke, amyotrophic lateral sclerosis, and dementia. To date, however, the therapeutic potential and safety of G-CSF for chronic TBI has yet to be determined. The aim of the present study was to identify the safety and potential effect of G-CSF for patients with TBI.

## 2. Methods

### 2.1. Patient Selection and Clinical Evaluation

This study was approved by the institutional review board of Tzu Chi General Hospital, Hualien, Taiwan. All methods were carried out in accordance with the relevant guidelines and regulations. A total of 38 chronic TBI patients in the study were interviewed over a period of two years (May 2016–March 2018) and had successfully completed the rounds of G-CSF treatment. We hope this preliminary report helps to explore the safety and potential efficacy of G-CSF for chronic TBI.

Our patient inclusion criteria were as follows: (i) they had suffered the TBI event at least 6 months before the study; (ii) they were between 40 and 80 years old; and (iii) their disability symptoms were stable without significant improvement under regular rehabilitation within two months before and after enrolment. Our patient exclusion criteria were as follows: (i) there was contraindication for the administration of G-CSF; (ii) they had unstable underlying medical comorbidities (ex: DM and hypertension); and (iii) their white blood cell count was >75,000/μL after G-CSF administration.

G-CSF (Cat. No. 000670; Filgrastim Injection M300; Kirin, Tokyo, Japan) is available as a commercial product in the form of a colorless fluid (0.028 mg of polysorbate, 35 mg of D-mannitol, 0.42 mg of acetic acid, with sodium hydroxide). G-CSF was administered through a subcutaneous injection. The patients received six rounds of three daily G-CSF doses at 10 μg/kg/day. Patients were allowed 3 weeks of rest between these rounds; thus, the six rounds of injections were administered within a 6-month period.

The primary outcome measures were the modified Rankin scale and the Karnofsky Performance Score and the secondary outcome measures are the laboratory measures. The modified Rankin scale is a widely used measure to report global disability. The grading ranges from category 0 to 6, with 0 indicating no symptoms and 6 being death [11]. Given its high validity and inter-rater reliability, it has been applied in evaluating patients with neurological diseases, such as TBI and stroke. The Karnofsky Performance Score is also a clinician-reported scale to evaluate general performance status on a scale of 0–100 with intervals of 10. A score of 100 indicates normal function with no disease sequel and death is graded a score of 0 [12]. Before the G-CSF treatment was administered, the patients were evaluated according to the modified Rankin scale and Karnofsky Performance Score. To ensure that the study could identify the clinical outcome of G-CSF treatment, patients did not change their medication during medical follow-ups. After the six rounds of G-CSF treatment in 6 months, all patients were evaluated. Laboratory measures included the leucocyte count (with a differential count), liver function, renal function, and glucose levels.

### 2.2. Statistical Analysis

Student’s paired *t*-test was used to compare the clinical measures and laboratory values of patients between and after G-CSF administration, with significance set at *p* < 0.05.

## 3. Results

The chronic TBI patients’ characteristics are summarized in Table 1. All 38 patients completed their 6-month G-CSF treatment period with regular follow-ups. Their comorbidity and medication lists included hypertension and diabetes mellitus, and the dose and regimen of medications had been stable in all participants before study enrolment. Before G-CSF administration, the modified Rankin scores revealed that around 27.4% of chronic TBI patients were at grade IV, 55.3% at grade III, and 18.4% were at grade II. After 6 months of G-CSF treatment, the modified Rankin scores significantly improved from 3 ± 1 pre-G-CSF to 2 ± 1 post G-CSF administration (mean ± SD, *p* < 0.05). The patients’ mean KPS also improved significantly from 45 ± 14 pre-G-CSF to 58 ± 14 post-G-CSF (mean ± SD, *p* < 0.01). White blood cell counts at baseline were 7316 ± 2426 μL (mean ± SD), but these increased significantly by around 6-fold at day 5 after G-CSF injection to 44,096 ± 14,236 μL. The band forms of leucocytes accounted for 27.4 ± 7.9% at day 5 of G-CSF treatment. A biochemistry study including liver and renal functions were within normal values during and after treatment.

There were no serious side effects related to the use of G-CSF during the study. Among the observed adverse effects, however, the most common included transient myalgia over the lower back (*n* = 2), diffuse soreness over four limbs (*n* = 2), and joint pain (*n* = 1). Mild dizziness, nausea, and gastrointestinal discomfort were also reported during the treatment period (within a 4-day round; *n* = 2). All symptoms subsided spontaneously or were quickly ameliorated following administration of nonsteroidal anti-inflammatory medications.

## 4. Discussion

To the best of our knowledge, this is the first study in which a standard dose of G-CSF (10 μg/kg) was used as a repeated chronic (over a 6-months period) treatment for patients with chronic TBI. After the final G-CSF injection round, the KPS of patients improved significantly, which suggests that G-CSF has potential benefits as a treatment for TBI at the chronic stage. Chronic G-CSF administration was also apparently safe as most adverse effects were mild and transient. (Safety)

Clinical studies in which G-CSF is used to treat neurological diseases are increasing. In most of these studies, G-CSF was administered as 5-day rounds to treat diseases such as stroke, amyotrophic lateral sclerosis, and dementia [13,14,15]. This common treatment regimen is likely applied following the method of clinical administration for bone marrow donors. It is usually well tolerated and safe with only minimal transient side effects, such as myalgia or joint pain [14]. In another study, two G-CSF rounds, each including 10 days of injections, were used to treat progressive amyotrophic lateral sclerosis and shown to be safe; although there was no significant clinical improvement after G-CSF treatment, the group injected with G-CSF showed amelioration of structural neural damage within 1 month [16]. This indicates that extended and repeated use of G-CSF for chronic neurological diseases might be feasible. A previous study also showed that higher serum levels of G-CSF in patients with TBI correlate with better outcomes at 6 months after injury [17].

Studies involving a rodent model of TBI have previously revealed the benefit of chronic G-CSF treatment [18,19]. The G-CSF-induced improvement in motor and cognitive disability results from the amelioration of neurodegeneration and loss of neuronal connectivity and plasticity [20]. In addition to increasing previously reduced dendrites and axons, administration of G-CSF also suppresses abnormally overexpressed excitatory synapses and the ensuing demyelination. These improvements in chronic TBI pathophysiology after G-CSF treatment could possibly explain our observation of improvement after G-CSF was used in patients with chronic TBI.

G-CSF has shown the ability to promote and maintain endogenous neurotrophic factors, such as BDNF, and thereby improve TBI [21]. This suggests that G-CSF could be a possible TBI treatment with its ability to cross the blood–brain barrier and exert beneficial effects on neural plasticity or preclude progressive neurodegeneration after TBI. In addition, G-CSF administration has been shown to increase and mobilize the incorporation of mesenchymal stem cells into the injured brain following TBI [22]. This evidence collectively indicates that G-CSF not only facilitates an increase in neurotrophic factors but also ameliorates the disability caused by TBI through integrated neurogenesis. Although we routinely used mannitol after G-CSF administration as a method to temporarily open the blood–brain barrier and facilitate the benefit of circulating stem cells, we did not test another group without mannitol use to identify the difference [23]. Thus, an additional study to confirm the benefit of using mannitol to increase the efficiency of stem cells or trophic factors crossing the blood–brain barrier is warranted.

While the benefit of using G-CSF as an acute or salvage treatment method has previously been reported in animal studies [24], the chronic use of G-CSF as a treatment for chronic TBI has rarely been investigated. Compared with the negative control group, Sheibani et al. [25] found that G-CSF used twice per day for 7 days in a TBI mouse model produced minimal benefits on cognitive performance with similar motor impairment. Another study also found that acute use of G-CSF did not ameliorate the size of contusion, brain edema, and glutamate concentrations in cerebrospinal fluid in a rat model of TBI [26]. In contrast, a recent study showed that 3 days use of G-CSF in post-TBI mice significantly improved both motor and cognitive disability at the 2-week follow-up; this behavioral improvement was attributed to increased neurogenesis in the hippocampus, increased recruitment of microglia and astrocytes, and upregulation of the neurotrophic factor BDNF [21]. Revealing the extended benefit of G-CSF during the time window and subacute phase after TBI, Toshkezi et al. [27] showed that G-CSF used in combination with stem cell factor might still be beneficial for treatment initiation about 2 weeks after TBI in a mouse model.

We acknowledge that our preliminary study has several limitations. First, this is a cohort study with a small case number, which significantly undermines the reliability of the effectiveness of G-CSF. However, we aimed to explore the safety of an old drug, G-CSF, with its off-label use for patients with chronic TBI. Future studies to include more patients and even another control group of patients to confirm the effectiveness is warranted. Second, we did not perform sub-group analysis to identify those patients who will possibly get better improvement from G-CSF. Last, but not least, both the modified Rankin scale and Karnofsky Performance Score are easy to administer but give only a very raw and general picture of the patient status without a specific description of patients’ neurological disability. Future studies should include more specific and comprehensive measures of the motor and cognitive status of the patients in order to clarify the areas that mostly benefitted from the treatment.

## 5. Conclusions

Although a 6-month period of monthly G-CSF administration has not previously been reported, our study provides preliminary evidence to support the safety of using G-CSF monthly over 6 months for the treatment of chronic TBI. A future study including a randomized and controlled trial of G-CSF will shed further light on the efficacy of chronic G-CSF use for improving neurological outcomes in patients with chronic TBI.

## Figures and Tables

**Table 1:** Demographics of the chronic traumatic brain injury patients (*n* = 38).

Age (range) at treatment	53 (16–81)
Age (range) at traumatic brain injury	52 (15–80)
Sex (male/female)	28/10
Hypertension	14 (37%)
Diabetes Mellitus	8 (21%)
Benign prostate hyperplasia	1 (3%)
Coronary artery disease	1 (3%)
